# Parkinsonism in GPi‐DBS for Dystonia; When to Suspect Degenerative Parkinsonism?

**DOI:** 10.1002/mdc3.13552

**Published:** 2022-09-08

**Authors:** B.E.K.S. Swinnen, M. Beudel, P.R. Schuurman, R.M.A. de Bie

**Affiliations:** ^1^ Department of Neurology and Clinical Neurophysiology Amsterdam UMC location University of Amsterdam Amsterdam The Netherlands; ^2^ Department of Neurosurgery Amsterdam UMC location University of Amsterdam Amsterdam The Netherlands

**Keywords:** deep brain stimulation, dystonia, GPi‐DBS, parkinsonism, Parkinson's disease

Bilateral GPi‐DBS is an established treatment for refractory dystonia.^1^ Stimulation‐induced parkinsonism is a frequent and often disabling side effect of GPi‐DBS.^2^ This parkinsonism mainly constitutes of bradykinesia and axial motor signs, and can hence phenotypically mimic Parkinson's disease (PD).^2^ GPi‐DBS stimulation‐induced parkinsonism improves with stimulation discontinuation.^2^ Chronic management mainly comprises stimulation parameter adjustments like decreasing the current/voltage or stimulating through more dorsal contacts.

We present a 69 year‐old patient with isolated segmental (ie cervical and oromandibular) dystonia since the age of 41, with familial dystonia occurrence (ie brother and son). At the age of 60 he underwent bilateral GPi‐DBS with good and sustained dystonia improvement. Since the age of 65 he progressively developed parkinsonism (Video 1.A). Initially, it was suspected to be a GPi‐DBS stimulation‐induced side effect. However, parkinsonism worsened upon stimulation discontinuation (Video 1.B). DAT‐SPECT imaging, performed because of symptom progression (Video 1.C), was abnormal and parkinsonism but not dystonia was levodopa‐responsive (Video 1.D). Whole exome sequencing revealed a variant of unknown significance in the GNAL gene, segregation analysis of which is still ongoing at the time of writing, but was otherwise unremarkable. Hence parkinsonism in this patient probably concerns idiopathic PD, or the recently described entity degenerative parkinsonism following longstanding cervical dystonia.^3^

**VIDEO 1:** Longitudinal assessment of parkinsonism. Evaluation at the age of 67 reveals appendicular bradykinesia with DBS ON (part A), worsening with DBS OFF (part B). Evaluation at the age of 69 demonstrates progression of bradykinesia and a parkinsonian gait (part C), both improving with levodopa treatment (part D), whereas dystonic features (ie lateroflexion of the head and jaw‐opening dystonia) did not improve.

In line with GPi‐DBS being an established PD treatment, parkinsonism in this case was partially masked by the anti‐Parkinsonian effect of GPi‐DBS.^4^ Therefore, off‐stimulation worsening of parkinsonism in GPi‐DBS for dystonia should be a warning for concomitant degenerative parkinsonism. In such instances, dystonia can be managed by maintaining effective (ventral) stimulation, whereas parkinsonism can be treated with dopaminergic medication.

## Disclosures

**Ethical Compliance Statement:** The authors confirm that the approval of an institutional review board was not required for this work. Written informed consent has been obtained from the patient. We confirm that we have read the Journal's position on issues involved in ethical publication and affirm that this work is consistent with those guidelines.

**Funding Sources and Conflicts of Interest:** The authors declare that there are no funding sources or conflicts of interest to report.

**Financial Disclosures for the Previous 12 Months:** The authors declare that there are no disclosures to report.

## Author Roles

(1) Patient care; (2) Video editing; (3) Manuscript: A. Writing of the first draft, B. Review and critique.

B.E.K.S.S.: 1, 2, 3A.

M.B.: 1, 3B.

P.R.S.: 3B.

R.M.A.dB.: 3B.
